# A New Technique for Localization of Parathyroid Adenoma: Infrared Thermal Scanning of the Neck

**DOI:** 10.7759/cureus.47977

**Published:** 2023-10-30

**Authors:** Adem Şentürk, Erhan Aysan

**Affiliations:** 1 Surgical Oncology, Sakarya Training and Research Hospital, Sakarya, TUR; 2 General Surgery, Yeditepe University Faculty of Medicine, Istanbul, TUR

**Keywords:** new technique, adenoma, scanning parathyroid, thermal, infrared

## Abstract

Many radiological techniques are used to locate the adenoma preoperatively in cases of primary hyperparathyroidism, but the location of many adenomas still cannot be detected. Since adenomas are hypervascular lesions, their temperature is high. Infrared thermal scanning can reveal local temperature differences in hypervascular lesions. The location of the adenoma could not be determined by preoperative radiological examinations in a 58-year-old male patient who was scheduled for surgery with the diagnosis of primary hyperparathyroidism. By infrared thermal scanning, a nearly 2°F higher temperature was measured in the inferior of the right thyroid lobe compared to the other perithyroidal regions. During the exploration, the adenoma was found at this point and removed. Infrared thermal scanning of the neck is promising as a new technique that can be used both preoperatively and intraoperatively to locate the adenoma in primary hyperparathyroidism cases.

## Introduction

Hypercalcemia is a serious clinical condition with high morbidity and mortality risks. The most common cause of hypercalcemia is primary hyperparathyroidism. Primary hyperparathyroidism often develops as a result of adenomatous hyperplasia in a single parathyroid tissue. While 80%-85% of pHPT is caused by a single parathyroid adenoma, 4%-5% of pHPT is caused by a double adenoma. Furthermore, 10%-15% of pHPT is caused by multigland hyperplasia and less than 1% by parathyroid cancer [1]. When hyperparathyroidism causes symptomatic or end-organ damage, parathyroidectomy is indicated. Besides, parathyroidectomy is indicated in cases with high serum parathormone (PTH) and calcium levels [2,3]. The only curative treatment for pHPT is surgery [1]. Since parathyroid adenomas are small and ectopic localization anomalies are common, determining the adenoma’s localization in the preoperative period is very important. Although ultrasonography (US) and sestamibi scintigraphy (MIBI) are the most commonly used techniques, computerized tomography (CT) and magnetic resonance imaging (MRI) are also preferred [1-6]. USG is the primary diagnostic modality because it is easily accessible and does not contain radiation, but its disadvantage is that it depends on the radiolog. Scintigraphy is routinely used for localization detection despite radiation exposure. For adenomas that cannot be localized with these two techniques, CT and MRI, which are more costly, are used [7]. Despite numerous imaging techniques, the success rate in locating parathyroid adenomas is still unsatisfactory [2].

Infrared thermometers are devices that measure thermal radiation emitted by objects. They are sometimes called non-contact thermometers to describe the device's ability to measure temperature from a distance [8]. Thermogram studies have revealed that neoplastic lesions have high levels of thermal emissions related to their hypervascularity [9,10]. Parathyroid adenomas are also hypervascular neoplastic lesions.

In this study, we presented a case with primary hyperparathyroidism who was indicated for parathyroidectomy, and the location of the adenoma could not be determined in a preoperative US, MIBI, MRI, or 4D TC scan. In this case, the location of the adenoma was successfully determined by an infrared thermometer, both preoperatively and intraoperatively scanning. 

## Case presentation

A 58-year-old man with primary hyperparathyroidism was admitted to the Yeditepe University Kosuyolu Ihtisas Hospital, Endocrine Surgery Clinic. He presented with significant fatigue, pain in his arms and legs, and recurrent renal stones. Laboratory evaluation revealed preoperative total serum calcium of 11.3 mg/dL (normal range 8.5-10.5 mg/dL), serum phosphorus of 2,3 mg/dL (normal range 2.5-4.5 mg/dL), intact serum PTH 145 pg/mL (normal range 10-65 pg/mL), vitamin D 35 ng/mL (normal range 20-80 ng/dL), alkaline phosphatase 120 U/L (normal range 25-100 U/L), albumin 4.5g/dL (normal range 3.4-5.4g/dL), 24-hour urine calcium 450 mg/day (normal range 100-400 mg/day) (Table 1). Liver and renal function tests were normal. The bone densitometry scan showed evidence of osteoporosis with osteopenia of the bilateral femur and right arm (T scores were -2.7, -2.7, and -2.2, respectively; the normal value of the T score is above -1). Renal US examination revealed enlargement of the pelvicalyceal system in both kidneys, with stones with a maximum diameter of 11 mm in the right kidney and a maximum diameter of 6 mm in the left kidney. 

**Table 1:** Laboratory findings

Test	Value	Reference range
Calcium (mg/dL)	11.3	8.5 to 10.5
Phosphorus (mg/dL)	2.3	2.5 to 4.5
Intact serum PTH (pg/mL)	145	10 to 65
Vitamin D (ng/mL)	35	20 to 80
Alkaline phosphatase U/L	120	25 to 100
Albumin (g/dL)	4.5	3.4 to 5.4
24-hour urine calcium (mg/day)	450	100 to 400

A physical examination of the neck was normal, and no palpable masses were detected. In the US examination, thyroid tissue size and echogenicity were normal. There was no evidence of a thyroid nodule or parathyroid adenoma. In early and late (120th minute), images of the MIBI examination and also the MRI were negative for parathyroid adenoma. 

The surgical exploration decision was made by the endocrine council. The patient was informed that the location of the parathyroid tissue was not found with preoperative radiologic tests, and neck exploration is necessary to perform. He was also informed that his neck would be evaluated with an infrared thermometer, and the data obtained from this application could be used during the operation. Written consent from the patient was obtained.

After the patient received general anesthesia and the thyroidectomy position was placed, a neck scan was performed with an infrared thermometer (Benetech Gm300®, China). Because of the inducing effects of local temperature, any energy devices or intraoperative nerve monitoring systems were not used in this operation. 

A thermal scan was made in the peri-thyroidal region, holding it at a distance of 3 cm from the skin surface. While the operating room ambient temperature was 87°F, the peri-thyroidal region temperature was 92°F. The temperature was measured at 94°F only in the right lower parathyroid region (Table 2). The maximum temperature (94.3°F) measured point was marked with a permanent pen, and the operation was started with a small Kocher incision. After the skin and subcutaneous tissue dissection, the temperature of the lower right parathyroid region was 95°F. After muscular tissues were dissected, the temperature of the same region rose to 97°F (Table 1). When the right thyroid lobe has deviated medially, an 11-mm-diameter parathyroid adenoma is found in the thyrothymic ligament, posterior side of the thyroid. A total excision was done. On the first postoperative day, the serum PTH level was 47 pg/ml, the Ca level was 8.9 mg/dl, and the phosphorus level was 2.6 mg/dl, and the patient was discharged without any signs of hypocalcemia. Histopathological examination was reported as parathyroid adenoma.

**Table 2:** Temperature values of the thyroid margins

Temperature measurement time	Up Right (°F)	Down Right (°F)	Up Left (°F)	Down Left (°F)
Before Incision Temperature	92	94	92	92
After Incision Temperature	95	97	95	95

## Discussion

Preoperatively determining the location of the adenoma in the preoperative period is directly related to the success, duration, and complication risks of the parathyroidectomy operation [11,12]. Although many radiological techniques are used for this purpose, surgeons have to undergo many operations to determine the location of the adenoma. The most frequently preferred imaging techniques are US and MIBI, and their sensitivity is between 57%-93% and 54%-93%, respectively [1-6].

The temperature at the tissue level is high in hypervascular lesions such as adenoma and carcinoma [12,13]. This is also true for parathyroid adenomas. The X-ray-based radiological imaging technique that shows the temperature values of the tissues is called a thermogram. Since the 1970s, thermograms have been used to determine the location of hypervascular lesions and also evaluate their malignancy potential, especially breast masses and thyroid nodules. In these studies, the thermal map of the whole organ is obtained from the images of X-ray-based devices, and temperatures are shown as color scales, not as numeric values. The red-colored areas in the images are commented on as malignant masses. A higher temperature increase was detected in thermograms in breast and thyroid cancers than in normal cells [13]. Unsuccessful localization is generally observed in ectopically located parathyroid tissue. This may lead to an increase in complications such as unsuccessful surgery, prolonged surgery, unnecessary neck dissection, and vocal cord paralysis [14].

Nowadays, thermographic evaluation is not used in the clinical routine due to the high false positivity (warm mass but not malignant) rates. Furthermore, new high-tech devices, such as mammography, high-resolution US, etc., have been very sensitive to determining malignant masses [9,10,12].

There are a limited number of studies in the literature on the location of parathyroid adenomas by thermographic technique. The unique clinical series on this subject belongs to Samules et al. The author commented that parathyroid thermography is an incompletely tested procedure, and it may prove efficacious in the presurgical localization of parathyroid adenomas [13,15,16].

In recent years, infrared thermal imaging technology has been integrated into CT devices, and thermographic mapping studies with numeric values have been carried out [12]. These devices are expensive, and the patient is exposed to X-ray radiation during the imaging procedure. In our literature review, we did not find any study using mobile infrared numeric thermal measurement technology without containing X-ray radiation used for detecting parathyroid adenomas.

The infrared thermometer measures the thermal radiation emitted by the objects. The design essentially consists of a lens to focus the infrared thermal radiation onto a detector, which converts the radiant power into an electrical signal that can be displayed in temperature units after being compensated for ambient temperature. This permits temperature measurement from a distance without contact with the object to be measured [8,17].

In our patient with primary hyperparathyroidism, the location of the parathyroid adenomas was not detected in the preoperative US, MIBI, or MRI. With an infrared thermometer, we detected a temperature of at least 2°F higher in the lower pole posterior of the thyroid right lobe than in the other peri-thyroidal locations. We marked this point before the incision. During the neck dissection, the temperature in the marked location increased even more as we passed each layer, but there was no significant change in other peri-thyroidal locations. It means that when the tissue thickness between the device and the adenoma decreased, the value of the measurement of the temperature became more reliable. This situation also reveals that infrared thermal scanning during surgery is useful in cases where the location of the parathyroid adenoma is undetermined by the preoperative imaging techniques.

Because many thyroid nodules have a hypervascular structure, infrared thermometers may give false positive results, but this restriction also applies to US and MIBI. However, the advantage of the infrared thermometer is that it is a mobile device and can be used easily during surgery. When the lateral suspensory ligaments are cut, and the thyroid tissue mediates medially, false positivity is solved. The US cannot be used for the localization of intrathoracic adenomas. The use of a thermal camera enables the detection and processing of temperature differences in tissues. For this purpose, studies are being carried out to develop standardization protocols. Thermograms have advantages such as sensitivity, low cost, non-invasiveness, rapid applicability, and no harm to the patient [18].

## Conclusions

In this report, we observed in one case that infrared thermal scanning may be helpful in finding the localization of parathyroid adenoma. Infrared thermometers give promise as a new, inexpensive, easy, and either preoperative or intraoperatively used technique. The advantages of US are that it does not contain radiation, is non-invasive, and has a relatively low cost. US is relatively fast and portable compared to MIBI and allows bedside imaging. Disadvantages of US include its low sensitivity in the presence of multiple adenomas or in detecting ectopic glands and its poor air and bone penetration, which limits the detection of deeper glands around the trachea and esophagus in the neck and mediastinum.

Advantages of MIBI include the possibility of imaging a large area, reader expertise, ability to show ectopic and deep glands. MIBI can be used in patients who cannot receive iodinated CT contrast, who undergo 4D-CT due to contrast allergy or renal failure, and who have a lower radiation dose than 4D-CT. Disadvantages include the long duration of the technique and the resulting difficulty in patient compliance, the relatively high cost, and the possibility of false positivity in thyroid nodules.

## References

[1] Lo CY, Lang BH, Chan WF, Kung AW, Lam KS (2007). A prospective evaluation of preoperative localization by technetium-99m sestamibi scintigraphy and ultrasonography in primary hyperparathyroidism. Am J Surg.

[2] Grosso I, Sargiotto A, D'Amelio P (2007). Preoperative localization of parathyroid adenoma with sonography and 99mTc-sestamibi scintigraphy in primary hyperparathyroidism. J Clin Ultrasound.

[3] Aygün N, Uludağ M (2019). Surgical treatment of primary hyperparathyroidism: which therapy to whom?. Sisli Etfal Hastan Tip Bul.

[4] Berczi C, Mezõsi E, Galuska L, Varga J, Bajnok L, Lukács G, Balázs G (2002). Technetium-99m-sestamibi/pertechnetate subtraction scintigraphy vs ultrasonography for preoperative localization in primary hyperparathyroidism. Eur Radiol.

[5] Reeder SB, Desser TS, Weigel RJ, Jeffrey RB (2002). Sonography in primary hyperparathyroidism: review with emphasis on scanning technique. J Ultrasound Med.

[6] Kebapci M, Entok E, Kebapci N, Adapinar B (2004). Preoperative evaluation of parathyroid lesions in patients with concomitant thyroid disease: role of high resolution ultrasonography and dual phase technetium 99m sestamibi scintigraphy. J Endocrinol Invest.

[7] Mutlu F, Aka B.U (2023). Minimal invasive parathyroidectomy: single-center experience. Acta Med Nicomedia.

[8] Ray PP (2017). An IR sensor based smart system to approximate core body temperature. J Med Syst.

[9] Samuels BI (1972). Thermography: a valuable tool in the detection of thyroid disease. Radiology.

[10] Buchanan JB, Weisberg BF (1978). Breast thermography. J Ky Med Assoc.

[11] Song AU, Phillips TE, Edmond CV (1999). Success of preoperative imaging and unilateral neck exploration for primary hyperparathyroidism. Otolaryngol Head Neck Surg.

[12] Smit PC, Borel Rinkes IH, van Dalen A, van Vroonhoven TJ (2000). Direct, minimally invasive adenomectomy for primary hyperparathyroidism: An alternative to conventional neck exploration?. Ann Surg.

[13] Damiao CP, Montero JRG, Moran MBH (2020). Affiliations expand on the possibility of using temperature to aid in thyroid nodule investigation. Sci Rep.

[14] Zhao T, Xin Y, Shen H, Liu X, Wang J, Wang Q, Wei B (2020). Vocal cord paralysis due to ectopic parathyroid adenoma and function recovery: a case report and review of the literature. Endocr J.

[15] Samuels BI, Dowdy AH, Lecky JW (1972). Parathyroid thermography. Radiology.

[16] Samuels BI (1975). The Present Status of Parathyroid Thermography. JAMA.

[17] Qury N, Nisha P, Dhara G (2013). A comparative study of contact free infrared thermometer and mercury-in glass thermometer in neonatal temperature measurement with special reference to phototherapy. Indian J Sci Res.

[18] Politi S, Aloisi A Jr, Bartoli V, Guglietta A, Magnifica F (2021). Infrared thermography images acquisition for a technical perspective in screening and diagnostic processes: protocol standardized acquisition. Cureus.

